# Keratinase improves the growth performance, meat quality and redox status of broiler chickens fed a diet containing feather meal

**DOI:** 10.1016/j.psj.2022.101913

**Published:** 2022-04-07

**Authors:** Kai-Lin Xu, Guo-Xin Gong, Miao Liu, Lu Yang, Ze-Jing Xu, Si Gao, Meng-Yi Xiao, Tao Ren, Bing-Ji Zhao, Mahmoud M. Khalil, Ling Zhao, Lv-Hui Sun

**Affiliations:** ⁎ Hubei Hongshan Laboratory, College of Animal Science and Technology, Huazhong Agricultural University, Wuhan 430070, China; † College of Life Science and Technology, Huazhong Agricultural University, Wuhan 430070, China; ‡ Demonstration Center of Hubei Province for Experimental Animal Science Education, Huazhong Agricultural University, Wuhan 430070, China; § Wuhan Technology Institute of Industrial Holding, Wuhan 430019, China; # Monogastric Research Centre, School of Agriculture and Environment, Massey University, Palmerston North 4442, New Zealand

**Keywords:** keratinase, feather meal, performance, meat quality, broilers

## Abstract

The objective of this study was to assess the effects of dietary supplementation of keratinase on the production of broilers fed a diet containing feather meal. A total of 162 1-d-old Cobb 500 male broiler (n = 9 cages/diet with 6 chicks/cage) were randomly allocated to 3 dietary treatments. The broilers were fed a corn-soybean-feather meal based diet (**BD**), or BD supplemented with keratinase at 100,000 or 200,000 U/kg for 6 weeks. Compared to the control, dietary supplementation with 200,000 U/kg keratinase increased (*P* < 0.05) body weight gain (3.6–4.3%) and reduced feed conversion ratio (2.4–5.6%) during the various experimental periods, and also improved (*P* < 0.05) apparent total tract digestibility of ash and calcium by 45.0% and 8.8%, respectively. Meanwhile, dietary supplementation of keratinase at 100,000 U/kg reduced (*P* < 0.05) the drip loss (29.2%), while 200,000 U/kg keratinase supplementation increased (*P* < 0.05) the pH value (1.6%) at 45 min and decreased (*P* < 0.05) the lightness (L* value; 13.6%) and drip loss (22.1%) of pectoral muscle. Moreover, dietary supplementation of keratinase at both levels of 100,000 and 200,000 U/kg increased (*P* < 0.05) Glutathione peroxidase activity (82.5–87.5%) and decreased the Malondialdehyde concentration (14.5–18.3%) in the pectoral muscle. In conclusion, dietary supplementation of keratinase at 200,000 U/kg can improve the performance, meat quality, apparent total tract digestibility of nutrients, and redox status of broiler chickens fed a diet containing feather meal.

## Introduction

The shortages and waste of feed resources are major constraints limiting the high productivity of poultry all over the world (Tsegaye et al., 2008; Duguma et al., 2017; Yin et al., 2019). Feathers are the mass-produced waste product of poultry production and can be processed into feather meal (Jagadeesan et al., 2020). A properly hydrolyzed feather meal contains approximately 85% crude protein, which can be used as a source of protein feed (McCasland and Richardson, 1966). Feather meal has the potential as a feed ingredient source in alleviating the problem of food competition between human and animals (Onifade et al., 1998). Moreover, poultry feathers exhibit a massive environmental threat, as they are disposed to the environment, which increase the need to recycle these feathers as a strategy to reduce or eliminate the environmental pollution caused by the poultry production (Tesfaye et al., 2017; Adetunji and Adejumo, 2018).

Feather meal contains approximately 90% β-keratin, which makes it poorly digested by most of the endogenous enzymes of broiler chickens, including trypsin, pepsin and the other protease (Tesfaye et al., 2017). Methods have been developed and applied to improve the digestibility of feathers meal. Generally, steam hydrolysis is a conventional processing method to increase the bioavailability of feathers meal (Moritz and Latshaw, 2001). Furthermore, enzymatic treatment is another strategy to improve the digestibility of feathers meal. Keratinase is a kind of protease with higher proteolytic activity than the most known proteases. Keratinase has the ability to hydrolyze a broad range of proteins including keratin, casein, collagen, and proteins with cysteine disulfide bonds (Gradisar et al., 2005; Huang et al., 2018). Application of keratinase as feathers-degrading enzymes has been shown to accelerate the digestion of feathers keratin in previously reported in vivo studies (Kim and Patterson, 2000; Riffel and Brandelli, 2002). Moreover, keratinase can improve the performance and digestibility of nutrients in pigs and poultry fed corn-soybean meal based diets (Wang et al., 2006; Wang et al., 2008; Wang et al., 2011; Huang et al., 2018). However, effects of keratinase on the production of broiler chickens fed a diet containing feathers meal were not studied.

Therefore, the current study was designed to evaluate the effects of keratinase supplementation on the performance, carcass traits, meat quality, metabolic rate, biochemistry and redox status of broiler chickens fed diets containing feather meal.

## MATERIALS AND METHODS

### Birds, Diets, Samples Collection and Analyses

The experiment was approved, conducted and supervised by The Institutional Animal Care and Use Committee of Huazhong Agricultural University, China. A total of 162 1-d-old male broiler chickens (Cobb 500) with similar average body weight were randomly allotted to 3 dietary treatment groups with 9 replicates of 6 birds each. The birds were fed a corn-soybean-feather meal based diet (**BD,** Table 1), or BD supplemented with keratinase (Wuhan Technology Institute of Industrial Holding, Wuhan, China) at 100,000 U or 200,000/kg. The dose of keratinase supplemented to the diet was based on previous studies (Odetallah et al., 2003). All the broilers were allowed free access to water and the designated diets for 6 wk. The body weight of the birds was measured at days 0, 21, and 42 and feed intake was measured weekly for the calculation of body weight gain (**BWG**), feed intake (**FI**), and feed conversion ratio (**FCR**). Mortality was recorded throughout the trial. The total excreta collection of each cage was held. The total excreta collection of each cage was held by collection trays at days 36 to 42 to estimate apparent total tract digestibility (**ATTD**) of crude protein, gross energy, ash, calcium, and total phosphorus. During the collection period, recorded the feed intake and total excreta output of each cage daily. Mixed excreta daily and added the 10% hydrochloric acid. The mixed sample stored in airtight plastic containers at −20°C. After the 7 d collection, all the excreta from the same cage were mixed and stored at −20°C pending analysis. At the end of the trial, 9 broilers from each group (1 bird/cage) were selected based on the average body weight of treatment for the assessment of carcass traits after 8 h of feed deprivation as previously described (Ghanima et al., 2020). The broilers were euthanized by manual cervical dislocation (Stiewert et al., 2021). Following euthanasia, blood was collected from the jugular vein via venipuncture into tubes containing EDTA-Na_2_ and the serum was obtained by centrifugation at 1,000 g for 15 min at 4°C. Pectoral muscle samples were collected and stored at −80°C until further analyses.

**Table 1:** Composition and nutritional values of the basal diet.

Item	Day 1–21	Day 22–42
Ingredients (%)		
Corn	58.5	61.1
Wheat bran	1.1	1.1
Soybean meal	27.5	24
Feather meal^1^	4	4
Soybean oil	4.2	5.43
CaHPO_4_	2.1	2
CaCO_3_	1.3	1.1
Salt	0.2	0.2
Lysine	0.37	0.39
Methionine	0.20	0.18
Threonine	0.23	0.2
Vitamin and mineral premix^2^	0.3	0.3
Total	100	100
Nutrient composition^3^		
AME, MJ/kg	12.54	12.97
Crude protein^4^,%	21.65	20.32
Crude fat,%	6.58	7.80
Crude fiber,%	2.42	2.25
Ash, %	6.29	5.81
Ca, %	1.07	0.97
P,%	0.68	0.65
Available Phosphorus,%	0.49	0.47
Lysine,%	1.25	1.18
Methionine,%	0.50	0.46
Cysteine,%	0.43	0.41

1 The analyzed crude protein in the feather meal were 82.56%.

2 Vitamin and mineral premix provided/kg diet: iron, 100 mg; copper, 8 mg; manganese, 20 mg; zinc, 100 mg; selenium, 0.3 mg; iodine, 0.7 mg; retinyl acetate, 10280 IU; cholecalciferol 2280 IU; dl-α-tocopheryl acetate, 17.12 mg; menadione, 6.82 mg; thiamin, 2.28 mg; riboflavin, 5.68 mg; pantothenic acid, 12.25 mg; pyridoxine, 2.28 mg; niacin, 22.84 mg; biotin, 0.18 mg; folic acid, 1.12 mg.

3 Caculated.

4 The analyzed crude protein in the diets of Day 1–21 and Day 22–42 were 21.36% and 20.18%, respectively.

### Keratinase Activity Analysis

The keratinase activity was determined by keratin digestion method using 1.0% keratin in 0.05 M Tris–HCl buffer (pH 8.0) as substrate according to Cai et al. (2008). Briefly, the enzymatic activity of keratinase was determined by preparing a 1.0% solution of Tris-HCl (pH 8.0) with azo casein substrate. The test group: 1.0 mL of 200 U/mL keratinase solution was added to 1.0 mL of azo casein solution; the blank group: 1.0 mL of 200 U/mL keratinase solution was added to 1.0 mL of water and placed in a water bath at 40°C for 1.0 h. The reaction was terminated by adding 2 mL of 10% TCA, and the supernatant was centrifuged at 4,000 rpm and measured at 280 nm. The absorbance was measured at 280 nm, each 0.01 increase in absorbance was considered as 1 unit of enzyme activity.

### Apparent Total Tract Digestibility Analysis

The ATTD coefficients of nutrients were calculated following the formula as previously described: ATTD X = ([total X ingested-total X excreted]/total X ingested); where: X represents crude protein, gross energy, ash, calcium, and total phosphorus (Schiavone et al., 2017). Briefly, gross energy was measured by an adiabatic bomb calorimeter standardized (IKA C2000) with benzoic acid. Crude protein was measured following the Kjeldahl digestion method 984.13 (AOAC, 2019). Ash content was determined by burning samples in a muffle furnace by method 942.05 (AOAC, 2000). The calcium and phosphorus were analyzed by the permanganate titration method 990.03 (AOAC, 2000) and the colorimetric determination method 985.01 (AOAC, 2019), respectively.

### Meat Quality Analysis

The pectoral muscle was collected to measure meat quality (Huang et al., 2021). Briefly, the pH of the muscle was determined in triplicate at 45 min and 24 h after slaughtering by a pH meter (pH-Star, SFK-Technology, Denmark). Meat color (L* = lightness, a* = redness, and b* = yellowness) of the muscle was determined in triplicate at 45 min postmortem by a chroma meter (Minolta Camera, Osaka, Japan). The fresh meat samples were cut into shaped strips (1 × 1 × 3 cm) and weighed and then placed in a Whirl-Pak bag. After this, samples were reweighed after being hung in a 4°C cooler for 24 h to calculate drip loss. Then, another fresh meat samples were cut into shaped strips (1 × 1 × 3 cm) and weighed and sealed in a plastic bag, and then cooked in a water bath at 75°C for 45 min to calculate cooking loss.

### Biochemistry and Antioxidant Parameter Analysis

The activities of alanine aminotransferase (**ALT**), aspartate aminotransferase (**AST**) and alkaline phosphatase (**ALP**), along with concentrations of total protein (**TP**), albumin (**ALB**), blood urea nitrogen (**BUN**), and creatinine (**CREA**) in the serum were determined by an automatic biochemistry analyzer (Beckman Synchron CX4 PRO, Fullerton, CA). The total antioxidant capacity (**T-AOC**), activities of glutathione peroxidase (**GPX**), catalase (**CAT**), superoxide dismutase (**SOD**), and concentration of malonaldehyde (**MDA**) in the pectoral muscle were analyzed with a colorimetric method by the specific assay kits (A015, A005, A007, A001, and A003) purchased from the Nanjing Jiancheng Bioengineering Institute of China (Sun et al., 2016). Briefly, the T-AOC was measured by the principle that Fe^3+^ could be reduced to Fe^2+^ by an antioxidant system. The Fe^2+^ can bind with the phenanthroline complex that could be detected spectrophotometrically at 405 nm, as described by Lee et al. (1981). One unit of T-AOC was defined as a 0.01 increase in optical density by one gram of protein sample per minute at 37°C. The SOD activity was determined based on the utilization of tetrazolium salt for the detection of superoxide radicals generated by xanthine oxidase. One unit of SOD activity was defined as the activity that inhibits 50% dismutation of the superoxide radical (Wang et al., 2021). The GPX activity was measured according to the principle that it could promote the reaction of H_2_O_2_ with GSH to produce H_2_O and oxidized glutathione. One unit of GPX activity can be expressed by its enzymatic reaction rate and calculated by the consumption of 1 μmol/L GSH in one gram of protein per minute (Wang et al., 2021). The CAT activity was measured according to the Aebi method, a decrease in H_2_O_2_ at 405 nm for 1 min was observed to measure the activity of CAT. One unit of CAT activity was defined as the amount of CAT needed to decompose 1 mmol H_2_O_2_ per minute (Aebi, 1984). The absorbance at 535 nm as spectrophotometrically of the color derived from the reaction of MDA and thiobarbituric acid in acidic media was determined as the description of Janero (Janero, 1990).

### Statistical Analyses

For all parameters, prediction equations were conducted testing the linear and non-linear effects of keratinase dose, and the interactions as continuous variables. The full model equation was: y = a + bx + cx^2^, where y = response variable, a = intercept, b and c = coefficients, x =concentration of keratinase. Parameters estimates that were not significant in the model and were not included in a significant interaction were removed from the model and the estimates recalculated. Pen served as the experimental unit for all parameters measured. Significance was accepted at *P* ≤ 0.05 (Walk and Rao, 2020).

## RESULTS

### Growth Performance and Carcass Traits

Growth performance results are presented in Table 2. Compared with the control, dietary supplementation of keratinase at 200,000 U/kg increased (*P* < 0.05) BWG of broilers during the days 22 to 42 and 1 to 42 by 3.6 to 4.3%, respectively, as well as reduced (*P* < 0.05) FCR ratio during the days 1 to 21 and 1 to 42 by 2.4 to 5.6%. However, dietary supplementation of keratinase at 100,000 U/kg did not have any effect (*P* > 0.05) on the growth performance of broilers. Notably, carcass traits, including dressing percentage, semieviscerated percentage, eviscerated percentage, breast meat percent-age, leg meat percentage, feather weight, were not influenced by any of the dietary keratinase supplementations (Table 3).

**Table 2:** Effect of dietary keratinase on growth performance of broilers.^1^

Items	Control	100,000 U/kg keratinase	200,000 U/kg keratinase	*P* valuelinear	*P* valuequadratic
Day 1–21					
Body weight gain, g	743 ± 60	758 ± 63	785 ± 46	0.123	0.304
Feed intake, g	1055 ± 61	1066 ± 56	1051 ± 49	0.887	0.830
Feed conversion ratio, g/g	0.70 ± 0.21^b^	0.71 ± 0.26^b^	0.75 ± 0.24^a^	0.002	0.003
Day 22–42					
Body weight gain, g	1621 ± 58^a^	1635 ± 80^ab^	1680 ± 51^b^	0.061	0.151
Feed intake, g	2825 ± 173	2868 ± 132	2894 ± 92	0.625	0.342
Feed conversion ratio, g/g	0.58 ± 0.02	0.57 ± 0.02	0.58 ± 0.02	0.528	0.521
Day 1–42					
Body weight gain, g	2364 ± 04^a^	2393 ± 23^ab^	2465 ± 68^b^	0.042	0.116
Feed intake, g	3880 ± 225	3934 ± 181	3945 ± 117	0.443	0.717
Feed conversion ratio, g/g g/g	0.61 ± 0.01^b^	0.61 ± 0.01^b^	0.62 ± 0.01^a^	0.026	0.036

1 Values are means ± SD, n = 9. Means in a row with different superscript letters are different, *P* < 0.05.

**Table 3:** Effect of dietary keratinase on carcass traits of broilers.^1^

	Control	100,000 U/kg keratinase	200,000 U/kg keratinase	*P* valuelinear	*P* valuequadratic
Dressing percentage, %	89.4 ± 3.4	90.8 ± 0.4	89.8 ± 0.9	0.659	0.339
Semi-eviscerated percentage, %	83.6 ± 2.8	84.9 ± 1.5	84.1 ± 1.6	0.624	0.419
Eviscerated percentage, %	71.5 ± 3.1	72.3 ± 2.6	72.2 ± 1.7	0.560	0.779
Breast meat percentage, %	31.8 ± 3.7	29.7 ± 2.0	30.8 ± 1.8	0.485	0.260
Leg meat percentage, %	19.9 ± 4.5	20.9 ± 2.3	19.5 ± 2.9	0.792	0.637
Feather weight, g	146.5 ± 54.8	116.6 ± 13.9	130.4 ± 20.4	0.347	0.208

1 Values are means ± SD, n = 9.

### Apparent total Tract Digestibility of Nutrients and Serum Biochemistry

The results of the ATTD of nutrients are shown in Table 4. Compared with the control, dietary supplementation of keratinase at 200,000 U/kg increased (*P* < 0.05) the ATTD of ash and calcium by 45.0% and 8.8%, respectively. However, dietary supplementation of keratinase at 100,000 U/kg did not affect (*P* > 0.05) the ATTD of the analyzed nutrients. Notably, the biochemistry indexes, including ALT, AST, ALP, TP, ALB, BUN, and CREA, were not affected (*P* > 0.05) by dietary keratinase supplementation (Table 5).

**Table 4:** Effect of dietary keratinase on apparent metabolic rate of nutrients in broilers.^1^

	Control	100,000 U/kg keratinase	200,000 U/kg keratinase	*P* valuelinear	*P* valuequadratic
Gross energy, %	76.8 ± 5.4	77.7 ± 5.5	79.2 ± 2.3	0.280	0.556
Crude protein, %	63.4 ± 8.3	65.4 ± 8.23	66.8 ± 3.5	0.306	0.595
Ash, %	31.1 ± 10.9^a^	27.6 ± 15.9^a^	45.1 ± 13.1^b^	0.046	0.031
Calcium, %	69.0 ± 5.8^a^	74.4 ± 7.9^ab^	75.1 ± 4.4^b^	0.047	0.095
Phosphorus, %	34.4 ± 12.3	36.8 ± 9.1	36.0 ± 5.9	0.724	0.867

1 Values are means ± SD, n = 9. Means in a row with different superscript letters are different, *P* < 0.05.

**Table 5:** Effects of dietary keratinase on serum biochemical parameters of broilers^1^.

	Control	100,000 U/kg keratinase	200,000 U/kg keratinase	*P* valuelinear	*P* valuequadratic
ALT, U/L	5.73 ± 2.81	4.61 ± 3.06	4.19 ± 2.88	0.264	0.520
AST, U/L	353 ± 103	351 ± 69	354 ± 75	0.989	0.997
ALP, U/L	1763 ± 386	1745 ± 363	1458 ± 459	0.120	0.222
TP, g/L	26.3 ± 3.2	27.6 ± 3.3	27.0 ± 2.6	0.634	0.656
ALB, g/L	15.5 ± 1.1	16.4 ± 1.2	16.1 ± 1.2	0.338	0.276
BUN, mmol/L	0.56 ± 0.07	0.55 ± 0.05	0.58 ± 0.04	0.543	0.477
CREA, mmol/L	8.56 ± 2.01	9.89 ± 2.26	8.87 ± 1.56	0.817	0.332

1 Values are means ± SD, n = 9. ALB: albumin; ALP: alkaline phosphatase; ALT: alanine aminotransferase; AST: aspartate aminotransferase; BUN: blood urea nitrogen; CREA: creatinine; TP: total protein.

### Meat Quality and Redox Status of Pectoral Muscle

The influence of dietary treatments on meat quality of broiler chickens are demonstrated in Table 6. Compared with the control, dietary supplementation of keratinase at 200,000 U/kg increased (*P* < 0.05) the pH value of muscle (1.6%) at 45 min, and reduced (*P* < 0.05) the L* value (13.6%) and drip loss (22.1%). Meanwhile, dietary supplementation of keratinase at 100,000 U/kg decreased (*P* < 0.05) the drip loss of muscle (29.2%) than those of the control. The redox status results are presented in Table 7. Compared with the control, dietary supplementation of keratinase at 100,000 and 200,000 U/kg increased (*P* < 0.05) GPX activity by 82.5 and 87.5%, respectively, and decreased (*P* < 0.05) the MDA concentration by 14.5 and 18.3%, respectively, in the pectoral muscle.

**Table 6:** Effect of dietary keratinase on meat quality of broilers.^1^

	Control	100,000 U/kg keratinase	200,000 U/kg keratinase	*P* valuelinear	*P* valueQuadratic
pH _45min_	6.13 ± 0.08^a^	6.11 ± 0.06^a^	6.23 ± 0.06^b^	0.008	0.003
pH _24h_	5.91 ± 0.03	5.91 ± 0.06	5.90 ± 0.06	0.475	0.680
Meat color					
L* (lightness)	57.3 ± 7.5^b^	56.5 ± 3.5^b^	49.5 ± 5.4^a^	0.008	0.014
a* (redness)	10.7 ± 1.0	10.9 ± 1.7	11.7 ± 1.5	0.116	0.247
b* (yellowness)	6.22 ± 2.20	7.26 ± 1.62	6.37 ± 2.25	0.878	0.516
Drip loss, %	8.11 ± 0.50^b^	6.31 ± 0.95^a^	5.74 ± 0.89^a^	0.000	0.000
Cooking loss, %	16.7 ± 2.7	15.8 ± 1.7	17.0 ± 2.8	0.810	0.575

1 Values are means ± SD, n = 9. Means in a row with different superscript letters are different, *P* < 0.05.

**Table 7:** Effect of dietary keratinase on redox status of pectoral muscle in broilers.^1^

	Control	100,000 U/kg keratinase	200,000 U/kg keratinase	*P* valuelinear	*P* valuequadratic
T-AOC, U/gprot	14.9 ± 11.1	20.2 ± 12.0	16.3 ± 6.6	0.782	0.533
GPX, U/mgprot	8.0 ± 3.9^a^	14.6 ± 7.6^b^	15.0 ± 7.5^b^	0.043	0.073
CAT, U/mgprot	62.6 ± 14.3	67.5 ± 11.6	59.0 ± 8.9	0.512	0.320
SOD, U/mgprot	15.2 ± 4.9	15.9 ± 3.9	14.3 ± 4.0	0.652	0.712
MDA, nmol/mgprot	1.31 ± 0.18^b^	1.07 ± 0.26^a^	1.12 ± 0.18^a^	0.084	0.059

1 Values are means ± SD, n = 9. Means in a row with different superscript letters are different, *P* < 0.05. CAT, catalase; GPX, glutathione peroxidase; MDA, malonaldehyde; mgprot, milligram protein; T-AOC, total antioxidant capacity; SOD, superoxide dismutase.

## DISSCUSSION

The present study illustrated that dietary supplementation of keratinase can improve the growth performance of broiler chickens fed a corn-soybean-feather meal based diet. Specifically, dietary supplementation of keratinase at 200,000 U/kg significantly increased BWG during days 22 to 42 and 1 to 42, while reduced FCR during days 1 to 21 and 1 to 42. These outcomes are similar to previous reports, which showed that keratinase can improve the growth performance of swine and poultry (Wang et al., 2006; Wang et al., 2008; Wang et al., 2011; Huang et al., 2018). The beneficial effects of keratinase on the growth performance of broilers have been associated with its capacity to improve the digestibility of nutrients, such as crude protein, energy, amino acids and minerals (Wang et al., 2008; Wang et al., 2011; Eaksuree et al., 2016; Huang et al., 2018). However, the current study showed that only the digestibility of ash and calcium were improved when dietary keratinase supplemented at 200,000 U/kg. The digestibility of crude protein, energy and phosphorus were not affected by keratinase supplementation. This discrepancy might be due to the variations in keratinase doses, dietary structure, animal species and physiological stages (Giacobbo et al., 2021). The FCR was improvement by keratinase from days 1 to 21 and overall but not from days 22 to 42, which might be due to the crude protein concentration in the experimental diets is deficiency (21.65% vs. 23.0%) from the days 1 to 21 but adequate (20.32 % vs. 20.0%) from the days 22 to 42 than those of recommended by NRC (1994). The beneficial effects of keratinase supplementation might be eliminated by the former method due to the interference of the cecal microorganisms (Ravindran et al., 1999; Ravindran et al., 2017).

Carcass traits are economically important yield characteristics in farm animals. Similar to the previous studies (Giacobbo et al., 2021), the current study showed that the carcass traits of broilers were not affected by the dietary supplementation of keratinase. Additionally, activities of serum enzymes including ALT, AST, and ALP, along with concentrations of serum TP, ALB, BUN, and CREA have been well-documented as valuable biomarkers for the health status of animals (Oner et al, 2008). These parameters were not affected by the dietary supplementation of keratinase, which were in agreement with the previous studies (Jiang et al. 2020). These outcomes revealed that keratinase was nontoxic and safe to the broilers.

Another interesting finding of the current study is that meat quality of broilers improved by the dietary supplementation of keratinase. Specifically, dietary supplementation of keratinase at 100,000 U/kg reduced the drip loss, and dietary supplementation of keratinase at 200,000 U/kg increased the pH value of muscle at 45 min, along with decreased lightness (L* value) and drip loss than those of the control. Abnormally fast postmortem glycolysis lead to a rapid pH drop in muscle when temperature of carcass is still high, resulting in decreased water holding capacity and protein extractability in meat (Rathgeber et al., 1999). Therefore, the low pH value and high drip loss are important characteristics of pale, soft, exudative (**PSE**) meat (Rathgeber et al., 1999). Meanwhile, it has been reported that lightness had a positive correlation with PSE meat (Rathgeber et al., 1999; Chen et al., 2017). Therefore, keratinase increase the meat pH value and decrease lightness and drip loss indicated it improved the meat quality of broilers.

Previous studies showed that oxidative stress can speed up the drop in the pH of meat, and increase the lightness and drip loss of postmortem (Chen et al., 2017; Wang et al., 2017; Markovic et al., 2018; Wang et al., 2018). In this study, dietary supplementation of keratinase at both doses of 100,000 U/kg and 200,000 U/kg have a better redox status, indicated by higher GPX activity and lower MDA concentration, than those of the control. The better redox status in muscle by keratinase supplementation could be explained by 1) keratinase improved the digestibility of ash, which might improve the utilization of selenium; as a selenium-dependent enzyme (Sunde, 2021), GPX activity could be improved by the selenium utilization (Zhao et al., 2017); 2) keratinase can improve the hydrolysis of the proteins containing cysteine to release cysteine (Gradisar et al., 2005; Huang et al., 2018), a precursor of glutathione, thus increase the body synthesis glutathione (Dennis et al., 2019); 3) glutathione/GPX system plays an important role in protecting the cells against oxidative stress (Fu et al., 2007; Surai, 2020). Taken together, these findings could help explain that dietary supplementation of keratinase improved the meat quality relative to the control.

In summary, the present study revealed that supplementation of 200,000 U/kg keratinase can improve the growth performance, ATTD and meat quality of broilers fed diets containing feather meal. Moreover, the improved meat quality in broilers may be related to the better redox status contributed by the dietary keratinase supplementation. Overall, these findings suggest that supplementation of keratinase to the diet containing feather meal is a feasibly way to improve the production of broilers.

## Acknowledgments

We thank Hua Sun, Xin-Dong Xiang, Fang-Ke Wang, Xu Wang, and Yu-Xuan Huang for the technical assistance. This project was supported by Wuhan Technology Institute of Industrial Holding.

## Disclosures

K.-L.X., G.-X.G., M.L., L.Y., Z.-J.X., S.G., M.-Y.X., M.M.K., L.Z. and L.-H.S. declare they have no conflicts of interest; T. R. and B.-J.Z. are members of Wuhan Technology Institute of Industrial Holding.
